# PPARγ mRNA in the adult mouse hypothalamus: distribution and regulation in response to dietary challenges

**DOI:** 10.3389/fnana.2015.00120

**Published:** 2015-09-03

**Authors:** Yang Liu, Ying Huang, Syann Lee, Angie L. Bookout, Carlos M. Castorena, Hua Wu, Laurent Gautron

**Affiliations:** ^1^Division of Hypothalamic Research, Department of Internal Medicine, University of Texas Southwestern Medical CenterDallas, TX, USA; ^2^Department of Orthopedics, Tongji Hospital, Tongji Medical College, Huazhong University of Science and TechnologyWuhan, China

**Keywords:** transcription factors, *in situ* hybridization, mouse brain, confocal laser scanning microscopy, hypothalamus

## Abstract

Peroxisome proliferator-activated receptor gamma (PPARγ) is a ligand-activated transcription factor that was originally identified as a regulator of peroxisome proliferation and adipocyte differentiation. Emerging evidence suggests that functional PPARγ signaling also occurs within the hypothalamus. However, the exact distribution and identities of PPARγ-expressing hypothalamic cells remains under debate. The present study systematically mapped PPARγ mRNA expression in the adult mouse brain using *in situ* hybridization histochemistry. PPARγ mRNA was found to be expressed at high levels outside the hypothalamus including the neocortex, the olfactory bulb, the organ of the vasculosum of the lamina terminalis (VOLT), and the subfornical organ. Within the hypothalamus, PPARγ was present at moderate levels in the suprachiasmatic nucleus (SCh) and the ependymal of the 3rd ventricle. In all examined feeding-related hypothalamic nuclei, PPARγ was expressed at very low levels that were close to the limit of detection. Using qPCR techniques, we demonstrated that PPARγ mRNA expression was upregulated in the SCh in response to fasting. Double *in situ* hybridization further demonstrated that PPARγ was primarily expressed in neurons rather than glia. Collectively, our observations provide a comprehensive map of PPARγ distribution in the intact adult mouse hypothalamus.

## Introduction

Peroxisome proliferator-activated receptor gamma (PPARγ) is a ligand-activated transcription factor that was originally identified as a regulator of peroxisome proliferation and adipocyte differentiation (Issemann and Green, 1990; Dreyer et al., 1992; Kliewer et al., 1994; Tontonoz et al., 1994; Amri et al., 1995). PPARγ has been implicated in the cellular effects of endogenous fatty acids in peripheral metabolic tissues (Debril et al., 2001; Ahmadian et al., 2013). Furthermore, the thiazolidinedione drugs, which target PPARγ, are effective treatments for type 2 diabetes (Knouff and Auwerx, 2004; Knauf et al., 2006). A large body of evidence also suggests that functional PPARγ signaling occurs within the central nervous system (CNS). Specifically, PPARγ agonists coordinate the expressions of genes that are involved in neuronal fatty acid metabolism and the responses to brain injury (Heneka et al., 2000; Sundararajan et al., 2005; Tureyen et al., 2007; Quintanilla et al., 2008; Schintu et al., 2009; Zhao et al., 2009). PPARγ signaling has also recently been reported to be involved in the central control of glucose, feeding behavior and energy homeostasis (Diano et al., 2011; Lu et al., 2011; Ryan et al., 2011; Garretson et al., 2015). The hypothalamus has been implicated in the aforementioned actions of PPARγ on metabolic functions. Thus, the identification of PPARγ-expressing sites and cell types in the hypothalamus would greatly benefit our understanding of the mechanisms that underlie the neural control of metabolic functions.

However, reports on the expression level and distribution of PPARγ in the CNS have been contradictory. For example, early studies found that PPARγ protein and mRNA were either absent or expressed at low levels that were close to the limits of detection in the adult rodent brain (Issemann and Green, 1990; Braissant and Wahli, 1998; Cullingford et al., 1998; Wada et al., 2006). However, recent mRNA mapping studies have consistently demonstrated detectable PPARγ expression in the cortex, hippocampus, and olfactory bulb (García-Bueno et al., 2005; Bookout et al., 2006a; Ou et al., 2006; Victor et al., 2006; Gofflot et al., 2007; Sobrado et al., 2009; Lu et al., 2011; Liu et al., 2014). Evidence of significant PPARγ expression in other brain sites is rather limited. Three studies have detected PPARγ protein by western blot and immunohistochemistry in the midbrain (Breidert et al., 2002; Park et al., 2004; Carta et al., 2011). Additionally, several other studies have reported a significant amount of PPARγ in the whole hypothalamus or identified feeding-related hypothalamic nuclei using quantitative PCR (qPCR) and antibody-based techniques (Mouihate et al., 2004; Sarruf et al., 2009; Diano et al., 2011; Lu et al., 2011; Ryan et al., 2011; Long et al., 2014). Other studies described PPARγ in mediobasal hypothalamic neurons using *in situ* hybridization histochemistry (ISH) (Long et al., 2014; Garretson et al., 2015). Notably, the results of the latter studies are at odds with those of prior mRNA studies that showed minimal hypothalamic and midbrain PPARγ (Bookout et al., 2006a; Gofflot et al., 2007). Additionally, antibody-based studies have yielded highly inconsistent results and, therefore cast doubt on the specificities of the currently available antibodies. Specifically, PPARγ immunoreactivity has been found either in neurons (Park et al., 2004; Ou et al., 2006; Victor et al., 2006) or in a mixed population of neurons and unidentified glial cells (Moreno et al., 2004; García-Bueno et al., 2005; Sarruf et al., 2009; Zhao et al., 2009; Carta et al., 2011; Lu et al., 2011). Furthermore, these same studies have described PPARγ immunoreactivities in different cell compartments and furthermore disagree on the exact anatomical distribution of PPARγ immunoreactive cells within the CNS. In face of all of these aforementioned inconsistencies in the available literature, this study sought to evaluate the anatomical distribution of PPARγ–expressing brain cells using ISH and qPCR in a spatially resolved manner, with a special emphasis on the hypothalamus. Moreover, we studied hypothalamic PPARγ mRNA regulation in response to metabolic challenges.

## Materials and methods

### Animals and diets

Wild-type mice on a C57Bl/6 genetic background were obtained from the UTSouthwestern Medical Center Animal Resource Center. All mice used in our ISH study were young adult males (4- to 8-week-old) that were housed in a light-controlled (12 h on/12 h off; lights on at 7 a.m.) and temperature-controlled environment (21.5–22.5°C) in a barrier facility. The animals used for histology were fed *ad libitum* on a standard chow (Harlan Teklad TD.2016 Global). The above procedures were approved by the Institutional Animal Care and Use Committee of the University of Texas Southwestern Medical Center at Dallas.

### Tissue collection and preparation

On the day of sacrifice, mice were anesthetized with an overdose of chloral hydrate (500 mg/kg, i.p.) between 8:00 a.m. and 11:00 a.m. For RNAScope® ISH experiments, the brains were rapidly dissected from anesthetized mice and frozen on dry ice on a piece of aluminum foil. Using a cryostat, 14–16 μm brain sections were collected on SuperFrost slides and stored at −80°C. For the qPCR experiments, the samples were collected and prepared exactly as we have previously described (Bookout et al., 2006b; Lee et al., 2012).

### Chromogenic and fluorescent ISH

As summarized in Table 1, double-Z oligo probes were designed by the manufacturer (Advanced Cell Diagnostic). The tissue was processed for ISH following the manufacturer's instructions. Briefly, the tissue was fixed tissue in 10% formalin and pretreated with a protease-based solution (pretreatment 4) followed by hybridization at 40°C for 2 h. The probes were mixed using the recommended ratio of 50:1 by volume (c1:c2 probes). Signal amplification was achieved using either diaminodenzidine (chromogenic) or specific fluorophores (FITC and Cy5). Incubation with diaminodenzidine was 10 min. The sections were counterstained with either Fast-Red (Sigma #N3020) or DAPI (Vector Laboratories; H-1500). Totals of 4 and 5 mice were used for the chromogenic and fluorescent ISH, respectively.

**Table 1:** **List of reagents used for *in situ* hybridization and qPCR**.

**ISH RNAScope probes**				
**Gene name(s)**	**Accession #**	**Probe region**	**Manufacturer**	**Cat. #**
PPARγ	NM_0111463	170–1490	ACD	418821-c1
Rbfox3 (NeuN)	NM_001039167-1	1827–3068	ACD	313311-c2
dapB	EF191515	414–862	ACD	312037-c1
Cyclophilin B (Ppib)	NM_011149.2	98–856	ACD	313911-c1
**qPCR probes**				
	**Manufacturer**	**Cat. #**		
PPARγ	ABI	Mm01184322_m1		
Npy	ABI	Mm00445771_m1		
Avp	ABI	Mm00437761_g1		

Please note that the probes used in this study did not distinguish between the known isoforms of PPARγ.

### qPCR

Whole hypothalamus and brown adipose tissue (BAT) samples and laser-captured microdissected samples were collected according to methods detailed in two of our previously published studies (Bookout et al., 2006b; Lee et al., 2012). The BAT and hypothalamus samples were taken from a cohort of C57/Bl6 male mice of 8–9 weeks of age (*n* = 6; fed on Harlan Teklad #7001 chow) (Bookout et al., 2006b; Lee et al., 2012). Hypothalamic laser-dissected samples were obtained from another cohort of C57/Bl6 male mice of 6 weeks of age (*n* = 12/group) that included groups that were fed *ad libitum* on chow diet, fasted for 24 h, or fed on high fat diet for 14 weeks (42% kcal from fat, 0.2% cholesterol—Harlan Teklad TD.88137) (Lee et al., 2012). Real-time qPCR gene expression analysis was performed exactly as we have previously described (Lee et al., 2012). To avoid sample bias, whole hypothalamus and BAT RNA were diluted to similar levels as LCM derived RNA. All RNA samples were treated and analyzed in parallel. All data are expressed as the mean ± SEM. The statistical analyses were performed with GraphPad Prism 6 software. The data were analyzed with a Two-Way ANOVA (nutritional challenge vs. brain site) followed by Dunnett *post-hoc* tests. The probes are listed in Table 1. Graphs were made using GraphPad Prism 6.

### Digital images acquisition and analysis

Bright-field images were captured using the 10 ×, 20 ×, and 40 × objectives of a Zeiss microscope (Imager ZI) attached to a digital camera (Axiocam). A Zeiss Stemi 2000-C binocular miscroscope was also used to capture low magnification dark-field images of our diaminobenzidine-labeled tissues. Identical exposure parameters were applied to samples within each experiment. Fluorescent digital images were acquired with a 63 × oil objective of a Leica TCS SP5 confocal microscope (UTSouthwestern Live Cell Imaging Core). Scanning parameters included a pinhole of 1 and a line average of 8. Laser intensity, gain and offset were adjusted appropriately to improve the signal/background. We collected stacks up to 25 optical sections separated by a step of ~0.3–0.4 μm in a 512 × 512 pixel format. NIH Image J software was used to generate our final TIFF images with combined Z stacks. The Imaris 8.1.2 software was also used to produce orthogonal views of confocal z-stacks.

As done by us in the past (Gautron et al., 2010), the distribution of PPARγ in the brain was evaluated in three mice by considering the density of diaminobenzidine-labeled cells per brain region independently of its surface (Table 2). Densities were subjectively determined by visual inspection of brain sections as follows: very low, ±; low, +; moderate ++; high +++. Our results are meant to provide inherently qualitative estimates.

**Table 2:** **Relative densities of diaminobenzidine-labeled cells per brain region (outside of the hypothalamus)**.

**Brain regions**	**ISH signal strengths**
**OLFACTORY REGIONS**	
AOB	+
Cl	++
CxA	++
DTT	++
En	++
EPl	±
Gl	+
GrO	++
IPl	++
LOT	+
Mi	+++
Pir	+++
VTT	++
**VENTRICULAR SYSTEM**	
3Vep	++
Cc	±
ChP	+
LV	+
**VENTRAL STRIATUM**	
CPu	+
ICjM	+
LGP	+
VP	±
**SEPTUM AND HIPPOCAMPUS**	
DG	++
DS	++
Fields CA1-2	+
Field CA3	++
HDB	+
LSD	+
MS	+
TS	+
VDB	+
VS	++
**NEOCORTEX**	
Layers I-III	+++
Layers IV-VII	++
Ent	+
**CIRCUMVENTRICULAR ORGANS**	
VOLT	+++
SFO	+++
**THALAMUS**	
AM	++
APT	++
AV	++
CM	+
DLG	+
MG	+
MHb	+
PG	+
PV	++
PVA	++
Rt	++
STh	+
VPM	+
ZI	++
**MIDBRAIN**	
ML	+
MM	+
VTA	±
SNR	±
IPR	±
IPC	±
**AMYGDALA**	
BLA	+
BMA	+
BLP	+
BMP	+
PLCo	++
PMCo	++
**HINDBRAIN**	
3N	+
4N	±
5N	±
7N	+
10N	±
12N	++
Amb	±
CIC	±
Cu	+
DCDp	±
Dk	+
DLL	±
DR	±
ECIC	±
Gi	±
LSO	±
Me5	±
ILL	±
LC	±
LDTg	±
LPB	±
lRt	±
MdV	±
MnR	±
NTS	±
PAG	±
PCRtA	±
Pn	±
PnC	±
Pno	±
Pr5	±
Pr5VL	±
R	+
RtTg	±
SC	+
Sp5	±
SPO	±
SuVe	±
SuG	±
Tz	±
VCA	+
VCPO	±
VLL	±
**CEREBELLUM**	
P	+
GrL	++
MoL	+

Very low, ±; low, +; moderate, ++; high, +++. Abbreviations follow the nomenclature in the Franklin and Paxinos's Mouse Brain in stereotaxic coordinates (3rd edition): 3N, oculomotor nucleus; 4N, trochlear nucleus; 5N, motor trigeminal nucleus; 7N, facial nucleus; 10N, motor nucleus of the vagus; 12N, hypoglossal nucleus; 3Vep, ependymal layer of the 3rd ventricle; AM, anteromedial thalamic nucleus; Amb, nucleus ambiguus; APT, anterior pretectal nucleus; AOB, anterior olfactory bulb; Aq, aqueduct; AV, anteroventral thalamic nucleus; BLA, anterior basolateral amygdala nucleus; BMA, anterior basomedial amygdala nucleus; BMP, posterior basomedial amygdala nucleus; BLP, posterior basolateral amygdala nucleus; CA1-3, field of the hippocampus; cc, central canal; ChP, choroid plexus; CIC; central nucleus inferior colliculus; Cu, cuneate nucleus; Cl, claustrum; CM, central medial thalamic nucleus; CxA, cortex-amygdala transition; CPu, caudate putamen; DCDp, deep core of the dorsal cochlear nucleus; DG, dentate gyrus; DLG, dorsal lateral geniculate nucleus; Dk, nucleus of Darkschewitsch; DLL, dorsal nucleus lateral lemniscus; DR, dorsal raphe; DS, dorsal subiculum; DTT, dorsal tenia tecta; ECIC, external cortex inferior colliculus; En, endopiriform cortex; Ent, entorhinal cortex; EPl, external plexiform later; Gi, gigantoreticular nucleus; Gl, glomerular layer; GrO, granule cell layer; HDB, nucleus of the horizontal limb of the diagonal band; ICjM, island of Calleja, major island; ILL, intermediate nucleus lateral lemniscus; IPl, internal plexiform layer; IPC, caudal interpeduncular nucleus; IRt, intermediate reticular nucleus; IPR, rostral interpeduncular nucleus; LBP, lateral parabrachial nucleus; LC, locus coeruleus; LDTg, laterodorsal tegmental nucleus; LGP, lateral globus pallidus; LOT, nucleus of the lateral olfactory tract; LRt, lateral reticular nucleus; LSD, lateral septal nucleus; LSO, lateral paraolivary nucleus; MdV, ventral medullary reticular nucleus; Me5, mesencephalic trigeminal nucleus; ML, mediolateral mammillary nucleus; MG, medial geniculate nucleus; MHb, medial habenular nucleus; Mi, mitral layer; MM, medial mammillary nucleus; MnR, median raphe nucleus; MS, medial septal nucleus; NTS, nucleus of the solitary tract; PAG, periaqueductal gray; PCRtA, anterior parvicellular reticular nucleus; PG, pregeniculate nucleus; PLCo, posterolateral cortical amygdala; Pir, piriform cortex; PVA, anterior paraventricular thalamic nucleus; PMCo, posteromedial cortical amygdala; Pn, pontine nuclei; PnC, caudal pontine reticular nucleus; PnO, oral pontine reticular nucleus; Pr5, principal sensory 5; PV, paraventricular thalamic nucleus; Pr5VL, ventrolateral principal sensory 5; R, red nucleus; Rt, reticular thalamic nucleus; RtTg, reticulotegmental nucleus pons; SC, superior colliculus; SCh, suprachiasmatic nucleus of the hypothalamus; SFO, subfornical organ; SNR, substantia nigra reticular; Sp5, spinal trigeminal nucleus; SPO, superior paraolivary nucleus; STh, subthalamic nucleus; TS, triangular septal nucleus; Tz, nucleus of the trapezoid body; VCA, anterior ventral cochlear nucleus; VCPO, posterior octopus of the ventral cochlear; VLL, ventral nucleus lateral lemniscus; VDB, nucleus of the vertical limb of the diagonal band; VTT, ventral tenia tecta; VOLT, vascular organ of the lamina terminalis; SuVe, superior vestibular nucleus; VP, ventral pallidum; VPM, ventral posteromedial thalamic nucleus; VTA, ventral tegmental area; VTg, ventral tegmental nucleus; VS, ventral subiculum; ZI, zona incerta.

The distribution of PPARγ in the hypothalamus was also represented on drawings. Briefly, the outline of hypothalamic sections was drawn in Adobe Photoshop CS5.1 using images taken with a Zeiss Stemi 2000-C binocular microscope. Diaminobenzidine-labeled PPARγ-expressing cells were manually marked by individual dots.

In addition, we evaluated the hybridization strengths within select diaminobenzidine-labeled hypothalamic nuclei compared to the motor cortex (Bregma −1.70 mm). Brightfield images of non-counterstained sections were taken at 40 ×. Image J was used to select areas of interest containing labeling. To isolate diaminobenzidine-labeled areas from background, images were binarized. The mean integrated intensity was then calculated for each selected area and divided by the surface of the area. This was repeated in 2-3 sections per brain site in 3 different mice. The resulting ratio reflected the intensity of the ISH strengths.

The image editing software Adobe Photoshop CS5.1 was used to combine digital images into final plates with annotations. The size, contrast, brightness, vibrance, and sharpness of our images were adjusted for presentation purposes (as indicated in the legends), while careful attention was applied to ensure no adjustments showed anything that did not originally exist. Specifically, the adjustments were always uniformly applied to all of the images from the same experiment. DAPI-counterstained nuclei were converted to gray to achieve better contrast. Finally, the abbreviations were derived from Franklin and Paxinos's Mouse Brain in Stereotaxic Coordinates (third edition).

## Results

### General distribution of extra-hypothalamic PPARγ-expressing sites

**Figure 1:**
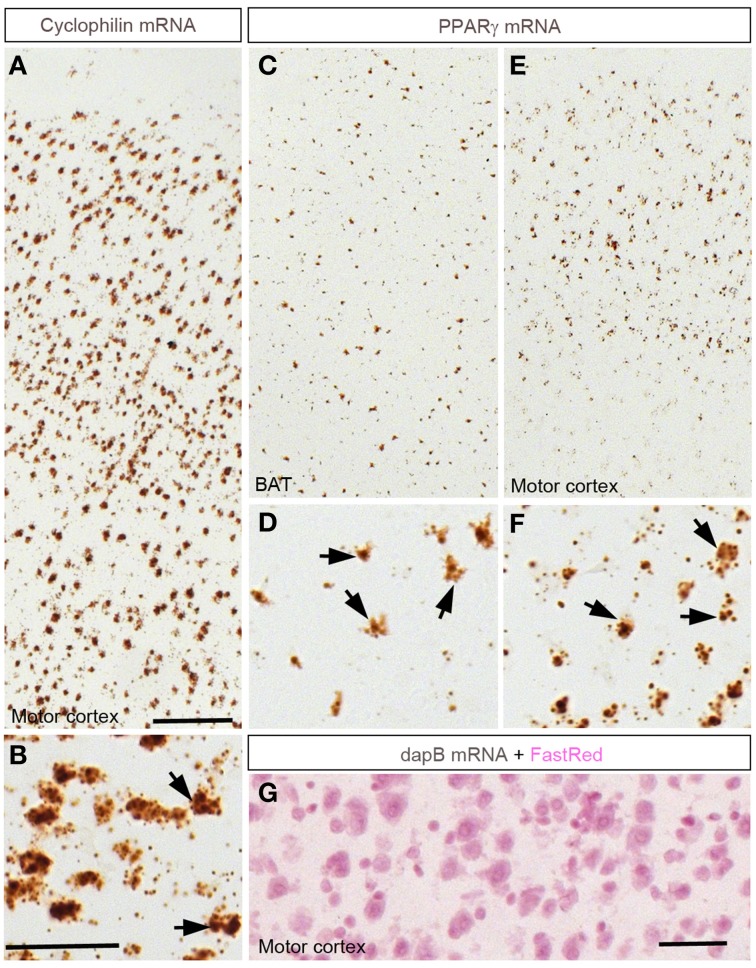
**Chromogenic detection of PPARγ in the mouse brain**. **(A,B)** Hybridization signals (brown) of Ppib in the motor cortex. As expected, Ppib was ubiquitously expressed across the entire brain including the cortex. **(C,D)** PPARγ hybridization signals in the brown adipose tissue (BAT). Presumptive adipocytes abundantly expressed PPARγ. **(E,F)** PPARγ hybridization signals in the cortex. Note that PPARγ expression was systematically higher in the outer layers of the neocortex. Black arrows indicate representative Ppib- or PPARγ–expressing cells. **(G)** Absence of signals in the neocortex hybridized with a probe against dapB and counterstained with Fast-Red. Minor adjustments in contrast or brightness were made uniformly. Abbreviations: BAT, brown adipose tissue; Scale bar in **(A,C,E)** is 120 μm; in **(B,D,F)** is 40 μm; in **(G)** is 50 μm.

The distribution of PPARγ-expressing cells was studied using a novel chromogenic ISH technique (RNAScope®) (Wang et al., 2012). The ISH signal was represented as a brown precipitate (diaminobenzidine) distributed in the cytoplasm immediately surrounding the cell nuclei (Figure 1). This approach generated virtually no background and therefore facilitated the identification of individual PPARγ-expressing cells. As a positive control, we attempted to detect the expression of cyclophilin mRNA in the brain (Figures 1A,B). Consequently, a very robust signal was ubiquitously detected across the entire mouse brain. Brown adipose tissue (BAT) was used as a positive control tissue for PPARγ expression. As anticipated, PPARγ was detected in many cells across the BAT in presumptive adipocytes (Figures 1C,D). Within the cortex, a robust PPARγ signal was also seen in many cells (Figures 1E,F). As a negative control, ISH was performed using a probe recognizing the prokaryotic gene dapB. Brain sections were completely devoid of a signal (Figure 1G). The distribution of PPARγ-expressing sites and the strengths of the hybridization signals are briefly described below and recapitulated in Table 2.

**Figure 2:**
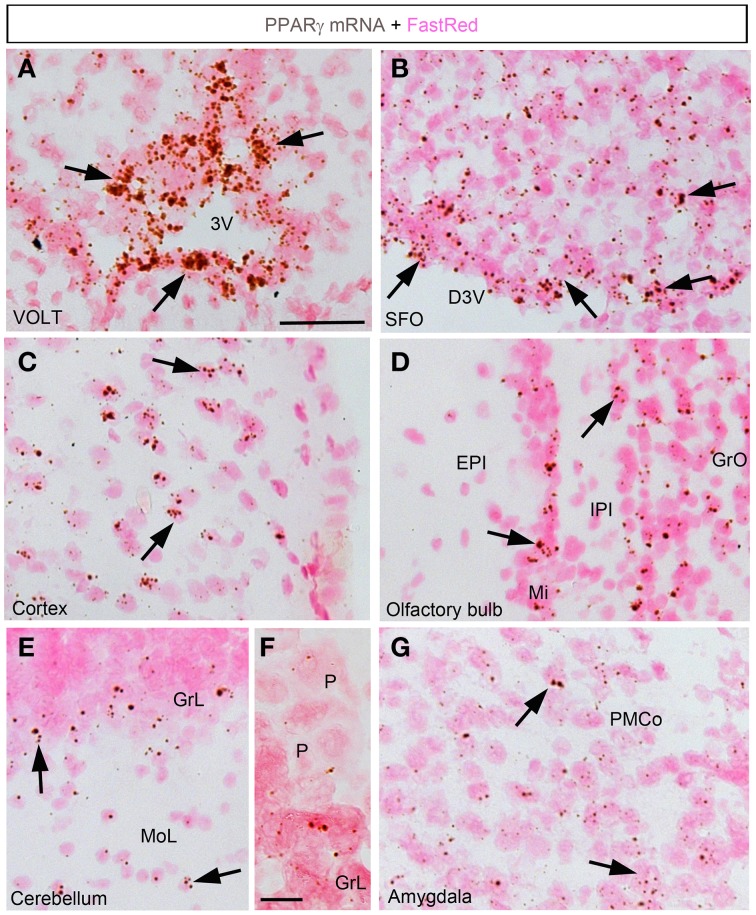
**Chromogenic detection of PPARγ in select brain regions**. **(A)** PPARγ hybridization signal (brown) in the VOLT. Note the presence of a very strong signal in cell forming an epithelium-like structure. **(B)** PPARγ hybridization signal in the SFO. PPARγ is present in both the ependyma covering the SFO and, to a lesser extent, in its ventromedial core. **(C)** PPARγ hybridization signals in the outer layers of the sensory cortex. **(D)** PPARγ hybridization signals in the olfactory bulb. **(E)** PPARγ hybridization signals in the cerebellum. The signal was concentrated in the granular layer. **(F)** Details of the hybridization signal at the edge of the granular layer. Please note low levels in Purkinje cells (P). **(G)** PPARγ hybridization signals in the amygdala were more prominent in the posterior and mediobasal parts. Minor adjustments in contrast or brightness were made uniformly. Tissue was counterstained with Fast-Red (pink). Abbreviations: 3V, third ventricle; D3V, dorsal third ventricle; GrL, granular layer; MoL, molecular layer; P, Purkinje cells. Other abbreviations can be found in the legend of Table 2. Scale bar in **(A–E,G)** is 40 μm. Scale bar in **(F)** is 12 μm. Arrows indicate representative positive cells.

**Figure 3:**
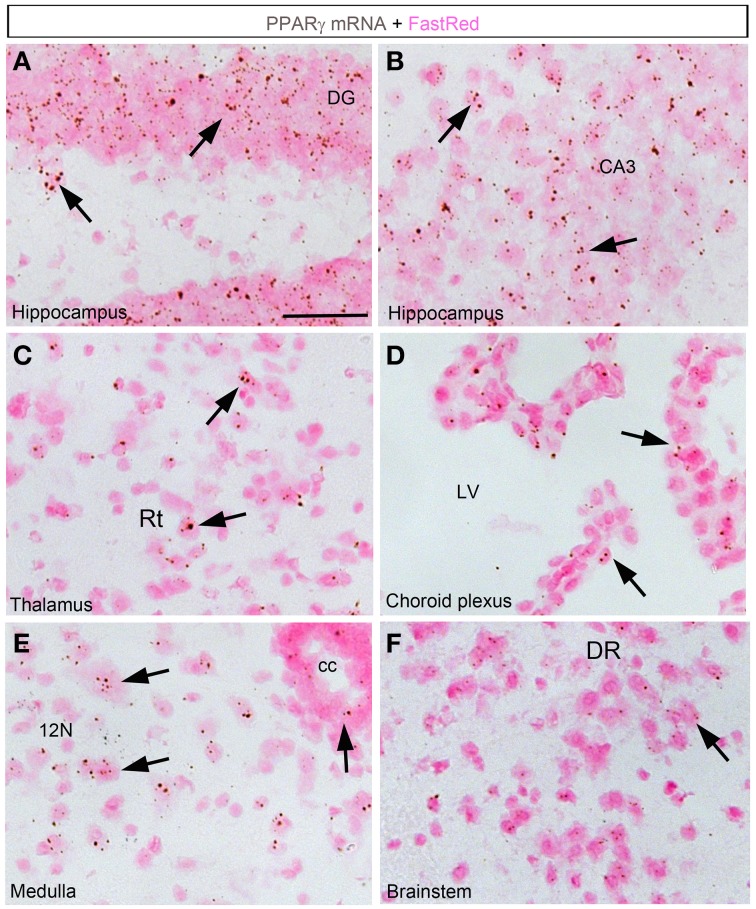
**Chromogenic detection of PPARγ in select brain regions**. **(A)** PPARγ hybridization signals (brown) in the dentate gyrus. Expression was seen in granular cells and, to a lesser extent, in the hilus. **(B)** PPARγ hybridization signals in the CA3 field of the hippocampus. Relatively abundant expression is seen across hippocampal neurons. **(C)** PPARγ hybridization signals in the thalamus. Scattered cells expressed moderate amount of PPARγ. **(D)** The choroid plexus of the 3rd, lateral and 4th ventricles contained moderate to low levels of PPARγ. **(E)** PPARγ hybridization signals in the medulla was generally very low. Nonetheless, motor neurons in the hypoglossal nucleus were positive. **(F)** Among other regions in the midbrain and pons, the dorsal raphe (DR) contained inconsistent and very low level of PPARγ. Minor adjustments in contrast or brightness were made uniformly. Tissue was counterstained with Fast-Red (pink). Abbreviations: cc, central canal; LV, lateral ventricle. Other abbreviations can be found in the legend of Table 2. Scale bar is 40 μm. Arrows indicate representative positive cells.

In the forebrain, the VOLT and subfornical organ (SFO) both exhibited very robust signals (Table 2; Figures 2A,B). The hybridization signal was particularly high in the ependymal layer bordering the lower edge of the organ of the vasculosum of the lamina terminalis (VOLT) and the SFO (Figures 2A,B). Less robust signals were also detected in the central capillary plexus of the VOLT and the ventromedial core of the SFO. Interestingly, PPARγ was not observed in other known circumventricular organs, including the median eminence and area postrema. The entire neocortex exhibited positive hybridization signals that increased intensity from the deepest to the most superficial cortical layers (Figures 1E,F, 2C; Table 2). PPARγ expression levels slightly varied among cortical areas differences, which ought to be related to differences in neuron structure and glial density (Elston et al., 2011). PPARγ was also expressed at high level in the olfactory bulb and most prominently in the mitral cell layer and granular cells (Table 2; Figure 2D). Other forebrain structures with moderate signals included a region encompassing the basomedial amygdala (Table 2; Figure 2G), select thalamic nuclei (Table 2; Figure 3C), and the choroid plexus (ChP) and ependymal cells of the cerebroventricular system (Figures 3D,E). Moderate levels of expression were also observed in the hippocampus and extended to the entire subiculum (Table 2; Figures 3A,B). Significant signal was also present in the globus pallidus and lateral portion of the caudate putamen (Table 2). In the rest of the forebrain, PPARγ expression was quite limited. Nonetheless, scattered cells exhibiting above-background signal were detected in the ventral pallidum, septal nuclei, islands of Calleja, and zona incerta, among a few other sites (Table 2). The hypothalamus will be separately described in a later section. Outside of the forebrain, positive cells generally expressed PPARγ at lower levels and were distributed in a more diffuse manner. In particular, a large number of sites in the midbrain and hindbrain exhibited a weak signal including many brainstem nuclei containing sensory or motor neurons. Among these sites, the hypoglossal nucleus (12N) displayed the most evident signal (Table 2; Figure 3E). In the other sites, including, but not limited to, the dorsal raphe (DR), the hybridization signals were very low and inconsistent (Table 2; Figure 3F). In the cerebellum, a moderate signal was distributed in the granular layer and, to a lesser extent, in the Purkinje cells and the cells of the molecular layer (Table 2; Figures 2E,F). Finally, it must be noted that PPARγ was never observed in the white matter.

### Detailed analysis of hypothalamic PPARγ expression and regulation

**Figure 4:**
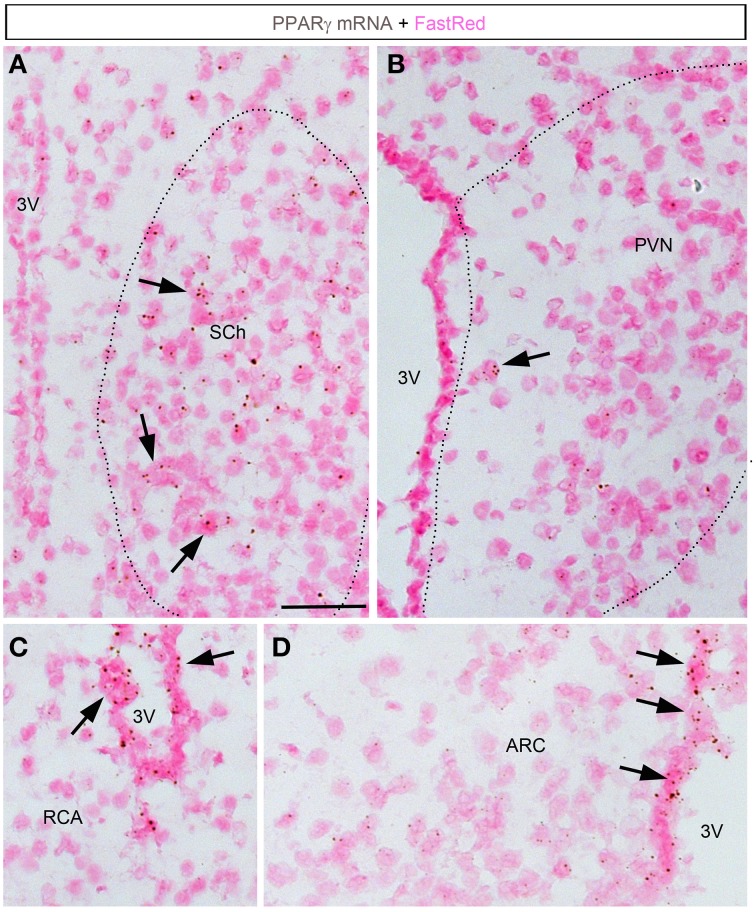
**Chromogenic detection of PPARγ in the mouse hypothalamus. (A)** PPARγ hybridization signals (brown) in the suprachiasmatic nucleus. Moderate expression was seen in scattered cells. **(B)** PPARγ hybridization signals in the paraventricular hypothalamus were very limited. **(C)** PPARγ hybridization signals in the retrochiasmatic area are observed in the mediobasal portion of the 3rd ventricle, but virtually absent from adjacent neurons. **(D)** The mediobasal portion of the 3rd ventricle contained positive ependymal cells. Neurons in adjacent ARC showed very little expression. Minor adjustments in contrast, brightness and sharpness were made uniformly. Tissue was counterstained with Fast-Red (pink). Abbreviations: 3V, third ventricle; ARC, arcuate nucleus of the hypothalamus; PVN, paraventricular nucleus of the hypothalamus; RCA, retrochiasmatic area. Scale bar is 40 μm. Arrows indicate representative positive cells.

**Figure 5:**
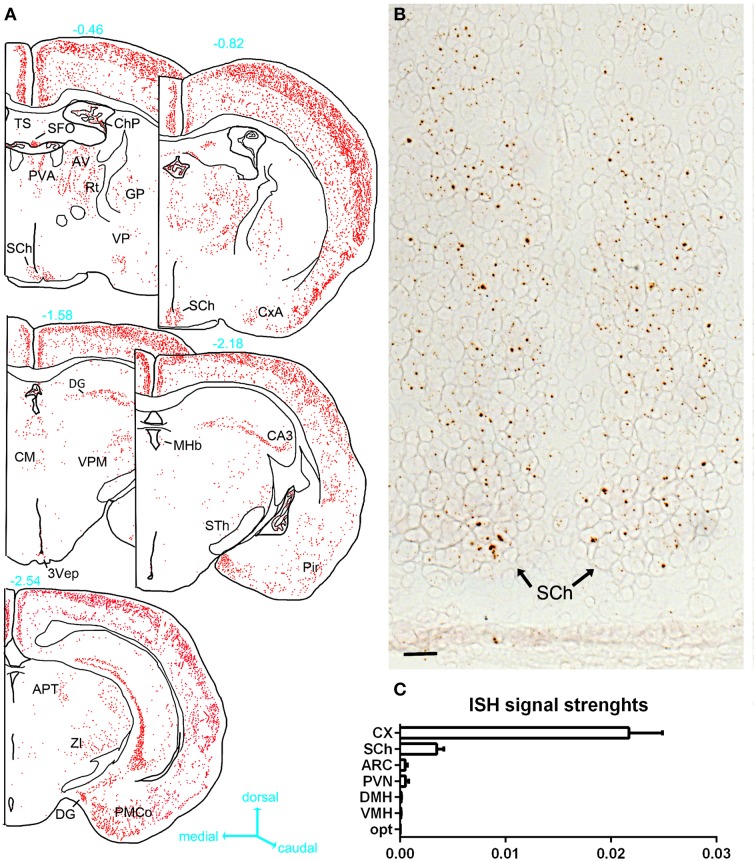
**(A)** Drawings of PPARγ hybridization signals in serial sections at the level of the hypothalamus. The estimated distance from the bregma (mm) is indicated in blue next to each section. Each red dot represents one identified diaminobenzidine-positive cell. Abbreviations can be found in Table 2. **(B)** PPARγ hybridization signals (brown) in the SCh (tissue was not counterstained). **(C)** Semiquantitative analysis of the ISH signals strengths in select hypothalamic nuclei compared to the cortex. Data represent mean ± S.EM. for three different animals. In the horizontal axis of this graph, arbitrary unit is used to express the ratio of the mean ISH signals/area (see methods for details). Scale bar is 50 μm. Abbreviations: Opt, optic chiasm; ARC, arcuate nucleus; CX, cortex; DMH, dorsomedial hypothalamus; VMH, ventromedial hypothalamus; SCh, suprachiasmatic nucleus of the hypothalamus.

ISH revealed very limited signals within the hypothalamus (Figures 4, 5). The suprachiasmatic nucleus (SCh) was the only hypothalamic nucleus in which the signal was clearly above background (Figures 4A, 5A,B). Using the chromogenic approach, cells with several brown dots per profile were commonly observed in the SCh (Figures 4A, 5B). The signal strengths were estimated to be roughly 5 times lower in the SCh than in the outer layers of the motor cortex (Figure 5C). The ependymal layer of the 3rd ventricle was also clearly positive for PPARγ (Figures 4C,D). The signal was more concentrated in the mediobasal portion of the 3rd ventricle (Figures 4C,D), an area that is enriched in tanicytes (Robins et al., 2013). In comparison, very little signal was contained within the rest of the hypothalamus including in the paraventricular nucleus of the hypothalamus (PVN), the retrochiasmatic area (RCA), and the arcuate nucleus (ARC) (Figures 4B–D, 5A). In the latter regions, PPARγ was expressed in scattered cells at very low levels that were close to the limit of detection. For example, the signal strengths were estimated to be roughly 45 times lower in the ARC than in the outer layers of the motor cortex (Figure 5C).

**Figure 6:**
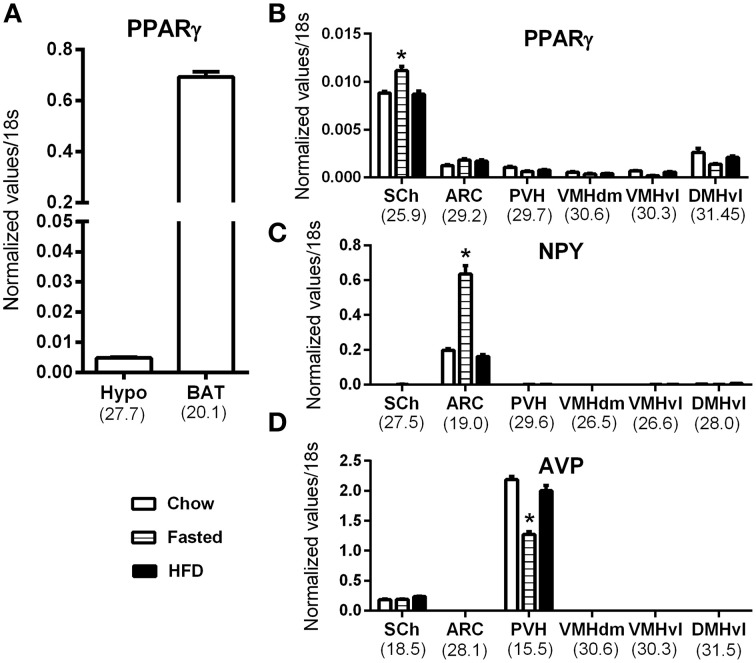
**QPCR analysis of PPARγ in the mouse hypothalamus**. **(A)** PPARγ mRNA expression in the whole hypothalamus (hypo) and brown adipose tissue (BAT) of chow fed mice. **(B)** PPARγ mRNA expression in laser-capture microdissected samples from various hypothalamic nuclei in mice submitted to nutritional challenges. **(C)** Neuropeptide Y (NPY) mRNA expression in laser-capture microdissected samples from various hypothalamic nuclei in mice submitted to nutritional challenges. **(D)** Vasopressin (AVP) mRNA expression in laser-capture microdissected samples from various hypothalamic nuclei in mice submitted to nutritional challenges. The value beneath each sample is the cycle number at threshold of the chow-fed group. Asterisks identify results that were significantly different from chow at *p* < 0.05. Abbreviations: ARC, Arcuate nucleus; BAT, Brown adipose tissue; DMHvl, dorsomedial nucleus of the hypothalamus, ventrolateral part; Hypo, hypothalamus; PVH, paraventricular hypothalamus; SCh, suprachiasmatic nucleus of the hypothalamus; VMHdm, ventromedial nucleus of the hypothalamus, dorsomedial part; VMHvl, ventromedial nucleus of the hypothalamus, ventrolateral part.

Because our results were at odds with two studies describing abundant ARC PPARγ (Long et al., 2014; Garretson et al., 2015), we next further investigated hypothalamic PPARγ mRNA by qPCR. As depicted in Figure 6, PPARγ mRNA expression was detected in the whole hypothalamus (Figure 6A). However, PPARγ expression in the hypothalamus was 140-fold lower than that in the BAT. To determine which hypothalamic nuclei accounted for the detected expression, we analyzed PPARγ expression in laser-capture microdissected hypothalamic nuclei according to the methods we have previously utilized (Bookout et al., 2006b; Lee et al., 2012). In agreement with our ISH results, the hypothalamic site that exhibited the highest level of expression was the SCh (Figure 6B). PPARγ was also detectable in the ARC, albeit at a low level (mean Ct ~29.2). In all other examined hypothalamic nuclei, PPARγ was near the limit of detection (mean Ct >30) (Figure 6B). Because studies reported an up-regulated PPARγ mRNA in the mediobasal hypothalamus of high-fat fed mice (Diano et al., 2011; Long et al., 2014; Garretson et al., 2015), we considered the possibility that the PPARγ expression level might be up-regulated by nutritional challenges. Therefore, we systematically compared samples from fasted, chow fed and high-fat fed mice. In the SCh, PPARγ was significantly upregulated in response to fasting (Figure 6B). However, in other nuclei, the levels of PPARγ expression were not regulated either by fasting nor high-fat feeding (Figure 6B). To further validate the sensitivity of our approach, we concurrently examined two marker genes that known to be abundantly expressed in identified feeding-related hypothalamic nuclei and to be regulated by nutritional challenges. As anticipated, neuropeptide Y (NPY) was only detected in the Arc and was up-regulated by fasting (Figure 6C). Vasopressin (AVP) was only found in the SCh and PVN (Figure 6D). AVP expression in the PVN was reduced by fasting.

### Identities of the PPARγ-expressing cells

**Figure 7:**
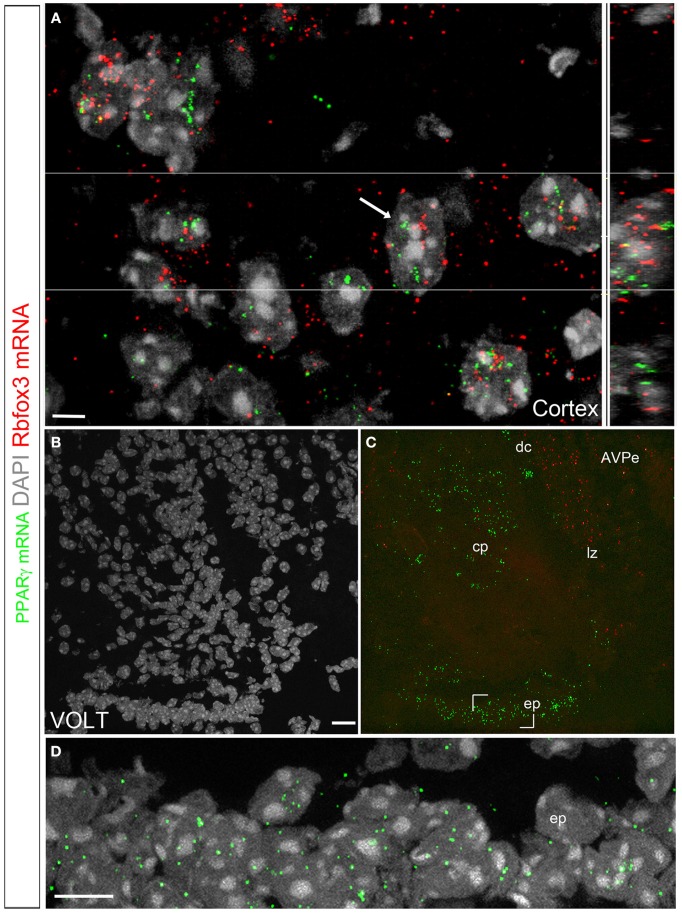
**Double fluorescent ISH for PPARγ and Rbfox3 in the mouse brain**. **(A)** DAPI counterstaining (gray) and hybridization signals for PPARγ (green) and Rbfox3 (red) in the neocortex. The white arrow indicates a representative cell with coexpression of both transcripts. The orthogonal view (Imaris) further illustrates the proximity of PPARγ and Rbfox3 mRNAs in the cell labeled with a white arrow. The above data indicate that PPARγ mRNA is primarily localized in neurons. **(B,C)** DAPI counterstaining (gray) and hybridization signals for PPARγ (green) and Rbfox3 (red) in the VOLT. PPARγ expression is apparent in the capillary plexus (cp), dorsal cap (dc), lateral zone (lz) and ependyma (ep). PPARγ and Rbfox3 were not coexpressed. **(D)** Details of the hybridization signals for PPARγ (green) in the VOLT ependyma. Adjustments in contrast, brightness, and vibrance were made uniformly. Abbreviations: AVPe, anterior ventral periventricular nucleus. Scale bar in **(A)** is 5 μm. Scale bar in **(B)** is 20 μm and applies to **(C)**. Scale bar in **(D)** is 12 μm.

We used a multiplex fluorescent approach to further characterize the identities of the PPARγ-expressing cells (Wang et al., 2012). The hybridization signal was represented by individual fluorescent dots that decorated the cytoplasm adjacent to the cell nucleus (Figures 7A–D). In the brain, signals were detected in the previously described PPARγ-expressing areas that included, among other examples, high levels in the cortex (Figure 7A) and VOLT (Figures 7B–D). The overall distribution of the observed fluorescent signals was directly comparable to that reported with the chromogenic approaches. Nonetheless, the fluorescent approach was less sensitive since areas previously described to contain very low levels of PPARγ mRNA did not contained fluorescent signals (i.e., feeding-related hypothalamic nuclei). Several elements suggested that the PPARγ-expressing cells in the parenchyma were primarily neurons. First, the distribution in well-defined parenchymal nuclei was suggestive of a neuronal localization. Second, based on the size (>10 μm), morphology (round with multiple nucleoli), and distribution (almost exclusively in the gray matter) of the DAPI-counterstained nuclei of the parenchymal PPARγ-expressing cells, the majority of these cells could be inferred to be neurons. Third, double fluorescent ISH revealed that PPARγ extensively colocalized with the pan-neuronal marker Rbof3 mRNA (also known as NeuN) (Figure 7A). For example, we observed nearly complete colocalizations between the PPARγ and Rbof3 mRNAs in the cortex (Figure 7A). Similar observations were made across the rest of the parenchyma. The VOLT, SFO, and the cerebroventricular system were the only sites containing PPARγ-expressing cells that did not exhibit colocalized Rbfox3 mRNA (Figures 7B–D). The arrangements of PPARγ-expressing cells in the latter structures were reminiscent of that of ependymocytes (Nehmé et al., 2012) (Figure 7D).

## Discussion

This study examined PPARγ mRNA expression in the intact adult mouse brain. Although many different types of cultured glial cells have been shown to express PPARγ (Bernardo and Minghetti, 2008; Dentesano et al., 2014), our data indicate that PPARγ is enriched in neurons in the intact brain. Contrary to our expectations, PPARγ mRNA was expressed at very low levels in all feeding-related hypothalamic nuclei. In contrast, PPARγ was found to be expressed in neurons at high to moderate levels in the entire neocortex, hippocampus, allocortex, and cerebellar cortex. The general pattern of PPARγ-expressing cells was reminiscent of those of other members of the PPAR family in the brain (Gofflot et al., 2007). Moreover, the enrichment of PPARγ in cortical and hippocampal neurons is consistent with the documented neuroprotective actions of PPARγ agonists in these neurons. PPARγ was also observed in select sensory and motor nuclei and in previously uncharacterized PPARγ-expressing sites, such as the SCh and circumventricular organs. We hope that our study will serve as a foundation for future research that aims to systematically delineate the physiological significance and transcriptional targets of PPARγ in both hypothalamic and extra-hypothalamic sites. The functional significance of our findings in the central regulation of metabolism is discussed below.

## Technical considerations

This study used ISH approaches to detect PPARγ mRNA in the mouse brain. In our hands, chromogenic and fluorescent ISH were all suitable for the mapping of PPARγ transcripts. Nonetheless, the chromogenic technique (RNAScope®) was the most sensitive of all. This technique also presented the advantage of generating almost no background and aided the identification of mRNA-expressing cells. Moreover, our ISH approaches present the advantage of not relying on the use of antibodies. This is important because peripheral immunoglobulins tend to accumulate in circumventricular organs and the ARC near the median eminence (Hazama et al., 2005; Yi et al., 2012), hence often resulting in the unwanted staining of these brain sites. Notably, the brain accumulation of immunoglobulins is exacerbated in metabolically-challenged animals (Hazama et al., 2005; Yi et al., 2012). Nonetheless, we must acknowledge one caveat regarding our findings. Although we don't expect the distribution of PPARγ to considerably vary with age, we cannot exclude age-related changes in PPARγ expression levels. In particular, animals used for our ISH mapping were younger than that those included in our qPCR study. This is was due to the necessity to expose our animals to dietary challenges (14 weeks on high-fat diet). Hence, we believe that additional ISH studies are warranted to examine PPARγ expression in the brain of older mice. Despite this caveat, the data obtained from qPCR of laser-captured hypothalamic sites strongly mirrored our ISH results both in terms of distribution and expression levels. Therefore, we are confident of the specificity and sensitivity our anatomical findings.

In contrast, we were not able to prove the specificity of PPARγ protein detection in the CNS using antibody-based techniques (data not shown). In particular, we tested without success two antibodies against PPARγ [Santa Cruz-7196 (H100) (Sarruf et al., 2009); Cell Signaling-2435S (C26H12) (Lu et al., 2011)]. Unfortunately, those antibodies detected several proteins other than PPARγ by western blot and, thus, might not be suitable for the detection of brain PPARγ by immunohistochemistry or western blot. Another antibody (Abcam ab191407) did not detect PPARγ by immunohistochemistry. In our opinion, our failure to identify a specific antibody against PPARγ is attributable to the fact that antibodies, and this particularly true of antibodies against receptors and signaling molecules, commonly produce unreliable staining (Saper and Sawchenko, 2003; Ivell et al., 2014). For example, it has been repeatedly shown that even antibodies that are widely used in the literature produce false positive results (Sim et al., 2004; Herkenham et al., 2011). This lack of selectivity might explain the discrepancies in the results of previously published immunohistochemical studies and our own data.

## Distribution and regulation of PPARγ in the hypothalamus

We observed moderate PPARγ expression in the SCh and, to our surprise, very little expression in feeding-related hypothalamic nuclei. These findings contrast with those of recent studies that linked hypothalamic PPARγ signaling to the regulation of energy metabolism using mouse genetics (Lu et al., 2011; Long et al., 2014). However, it must be stressed that the mouse lines that have previously been utilized to delete PPARγ from the CNS were not selective for the hypothalamus and consequently might have resulted in reduced PPARγ signaling in other brain regions. This situation applies to the synaptophysin-Cre mouse, which displays widespread Cre activity in both the hypothalamus and extra-hypothalamic sites in addition to peripheral sites such as the mesenchyme and alimentary, urinary and reproductive systems (see the recombinase activity at http://www.informatics.jax.org/allele/MGI:2176055). The same shortcoming applies to POMC-Cre mice, which have been demonstrated to display Cre activity during development in over 62 brain sites including many PPARγ-containing sites (e.g., the cortex, SCh, hippocampus) (Padilla et al., 2012). Two pharmacological studies also showed that the central administration of PPARγ agonists stimulated food intake (Ryan et al., 2011; Garretson et al., 2015). However, the brain site(s) responsible for these effects has not been elucidated and accumulating evidence suggests that PPARγ agonists may act on brain cells *via* other receptor(s) than PPARγ (Woster and Combs, 2007; Thal et al., 2011). More convincingly, the overexpression of PPARγ selectively in the adult rat hypothalamus using a viral approach resulted in altered feeding behavior and body weight (Ryan et al., 2011). The exact hypothalamic site(s) involved in these effects has not been characterized. Two prior studies suggested that ARC neurons may be important in the feeding effects of PPARγ (Long et al., 2014; Garretson et al., 2015). While it cannot be entirely ruled out that PPARγ signaling in the ARC may regulate feeding behavior, its expression level in this region was extremely low. Alternatively, based on our observations, it is tempting to hypothesize that the SCh and/or tanicytes might play a role in the neural control of metabolism *via* PPARγ. Although these sites are not traditionally considered to be critical to the regulation of energy balance, there is emerging evidence of their implications in the control of energy expenditure and locomotor activity (Bookout et al., 2013; Balland et al., 2014).

Notably, the presence of PPARγ in the SCh also implies that it is involved in the circadian control. While PPARγ has been linked to the regulation of circadian rhythms in peripheral tissues (Chen and Yang, 2014), to the best of our knowledge, the role of PPARγ in the SCh molecular clock machinery has not been well studied. It is particularly interesting that SCh PPARγ expression was upregulated by fasting, presumably due to increased circulating levels of fatty acids. Combined with the observation that food availability exerts a robust influence on clock genes expression in the SCh (Horikawa et al., 2005), our data suggest that PPARγ signaling in the SCh may serve as a link between metabolic cues and circadian rhythms. Overall, our observations call for a reappraisal of the role of hypothalamic PPARγ.

## Possible role(s) of circumventricular PPARγ in metabolism

Interestingly, high levels of PPARγ were identified in two circumventricular organs that have been implicated in the regulation of hydromineral homeostasis and drinking behaviors (Fitzsimons, 1998; Oka et al., 2015). This novel observation is important considering that PPARγ agonists that are used in diabetes treatment exert unwanted adverse effect on body fluid homeostasis that include an elevated rate of edema (Rizos et al., 2009). In addition to their actions on the kidney and cardiovascular system, our data support the idea that the circumventricular organs might be involved in PPARγ agonist-induced fluid retention. It should also be noted that the SFO is increasingly being recognized to regulate metabolic functions in addition to hydromineral homeostasis (Ferguson, 2014). Furthermore, an intact VOLT is required for the maintenance of a normal body temperature (Romanovsky et al., 2003). It is possible that the mouse lines that have previously been utilized to delete PPARγ from the CNS also targeted these areas and hence resulted in a metabolic phenotype. Considering that chylomicrons and very-low-density lipoproteins do not cross the blood-brain barrier, it is logical that PPARγ activity might peak in these two sites devoid of a blood-brain barrier. Hypothetically, the fatty acids released from the VOLT and SFO diffuse to the cerebrospinal fluid contained in the 3rd ventricle to be transported across the brain. If our hypothesis is correct, then PPARγ signaling in the SFO and VOLT might be a primary determinant of fatty acid uptake into the CNS. PPARγ signaling in the choroid plexus might also play a role in lipids metabolism in the brain.

### Conflict of interest statement

The authors declare that the research was conducted in the absence of any commercial or financial relationships that could be construed as a potential conflict of interest.
